# Anatomy, Descriptive and Surgical

**Published:** 1901-12

**Authors:** 


ANATOMY, DESCRIPTIVE AND SURGICAL.—By Henry Gray, F. R. S., St. George's Hospital, London. Thoroughly revised American from the 15th English edition. In one imperial octavo volume of 1246 pages, with 780 illustrations. Price, with illustrations in black, cloth, $5.50, net; leather, $6.50, net. Price, with illustrations in colors, cloth, $6.25, net; leather, $7.25, net. Lea Brothers & Co., Publishers. Philadelphia and New York.

For more than forty years this work has been almost without a rival. Ever since the appearance of the original edition it has easily held first rank among text-books on anatomy. However, the additions to our knowledge during recent years and the changes in the methods of teaching have rendered necessary a thorough revision of the text and the addition of numerous illustrations. The anatomists having charge of this important task have given the text a thorough going over and made many changes. For example, the section on embryology has been rewritten, and sixty or more new illustrations have been added. The section on the brain, cord, nervous system and viscera have also been much enlarged and improved. The work is much better for having undergone the revision, and will, without doubt, continue to be the standard text-book on anatomy for many years to come. R.

